# AI-Based Assessment of Non-Technical Skills in Prehospital Simulations: A Comparative Validation Study

**DOI:** 10.3390/healthcare13233121

**Published:** 2025-12-01

**Authors:** Masanori Mitsuhashi, Yumika Akiba, Misato Saitou, Kensuke Suzuki, Satoo Ogawa, Tomohiko Masuno

**Affiliations:** 1 Department of Emergency Medical Science, Faculty of Medical Science, Nippon Sport Science University, Yokohama 227-0033, Japan; k-suzuki@nittai.ac.jp (K.S.); masuno@nittai.ac.jp (T.M.); 2 Emergency and Disaster Medicine, Graduate School of Medical and Health Science, Nippon Sport Science University, Yokohama 227-0033, Japan; 25rma03@nittai.ac.jp (Y.A.); 25rma06@nittai.ac.jp (M.S.); 3 Department of Emergency Medical Science, Nippon Sport Science University, Tokyo 158-8508, Japan; stream@nittai.ac.jp

**Keywords:** non-technical skills, prehospital, simulation-based education, paramedic students, emergency medical services, artificial intelligence, Gwet’s AC2, Bayesian analysis, debriefing

## Abstract

**Background/Objectives**: Assessing non-technical skills (NTSs) in prehospital care is susceptible to rater subjectivity. While Artificial Intelligence (AI) can be used to score conversation transcripts, it emphasizes formal linguistic features, whereas humans integrate scene context, leading to potentially divergent evaluations. We examined the validity of NTS assessments generated by AI (ChatGPT-4o) from prehospital simulation data by comparing them with ratings from paramedic faculty. We hypothesized that AI-based ratings would provide evaluations of team NTSs that are comparable to faculty ratings and would enable us to describe the direction and magnitude of score differences between AI and faculty across the five NTS domains. **Methods**: Sixty-four first-year paramedic students performed 5 min prehospital scenarios. Five NTS domains were scored independently by AI and faculty using a three-level rubric (5, 3, or 1 point per domain): (i) communication and interpersonal manner, (ii) order and completeness of information gathering, (iii) detail of follow-up questioning, (iv) context-appropriate actions, and (v) time management. Score differences were analyzed with Wilcoxon signed-rank tests with Holm correction and Bayes factors (BF10). Agreement was quantified with weighted Gwet’s agreement coefficient 2 (AC2). **Results**: Three domains—communication, context-appropriate actions, and time management—showed significant differences (*p* < 0.001), with strong evidence for differences (BF10 > 22); median differences favored AI. Evidence of a difference was insufficient for the other two domains. Across all domains, agreement remained below the prespecified substantial threshold (AC2 ≥ 0.60). The primary hypothesis was not supported. **Conclusions**: In prehospital simulations, AI-only NTS assessment is not yet an adequate substitute for human raters. Although AI evaluates linguistic aspects, its agreement with expert ratings was insufficient. Future work should evaluate hybrid approaches leveraging the strengths of both AI and human judgment.

## 1. Introduction

Prehospital rescue emphasizes not only technical skills such as airway management and cardiopulmonary resuscitation but also non-technical skills (NTSs)—smooth information transfer within the team, accurate situational judgment, and leadership. These NTSs are closely related to what are often referred to as “soft skills”—interpersonal communication, teamwork, time management, problem solving, and empathy—however, in emergency medicine, they are formalized as observable behaviors that support safe team performance [1,2]. In the prehospital setting, crowd movement and hazards at the scene are constantly changing, information is fragmentary, and communications are easily interrupted. Under constraints of limited personnel and equipment, multiple professions must respond immediately and coordinate, and the team must instantaneously align situational awareness, role assignment and chain of command, and the triage-based priorities for rapid transport. Furthermore, prompt communication that reassures confused patients and their families constitutes an essential foundation supporting prehospital care [3,4,5,6,7].

Within NTSs, simulation-based education has been established as a pedagogical method that faithfully reproduces real clinical settings and provides practical learning opportunities in a safe environment [8,9]. However, two challenges remain in evaluating educational outcomes. First, assessments depend heavily on faculty’s subjectivity, making it difficult to ensure inter-rater reliability and consistency. Second, instructor-provided feedback entails substantial time and personnel burdens, constituting a factor that impedes increases in the number of learners and the frequency of sessions [3,8].

To address these issues, the use of large language models (LLMs) has advanced rapidly in recent years. In OSCE-related tasks such as scoring post-encounter notes derived from clinician–patient communication, LLMs have demonstrated accurate and reproducible scoring and, in some contexts, performance comparable to human raters, although agreement varies by domain [10,11].

Pears et al. conducted a double-blind study with urology trainees comparing AI (GPT-4) and human feedback under blinded conditions after simulation, in which experts performed quantitative and qualitative evaluations based on transcribed, annotated text. The study authors elucidated complementary characteristics: AI showed notable strengths in enhancing information gathering, evidence-based accuracy, empathic responses, and adaptation of explanations; in comparison, humans were superior in the use of terminology, control of complexity, and fact-based identification [12].

However, the primary outcomes of the above previous study were the educational effects of feedback and learners’ acceptance; the authors did not test the validity of AI scoring or statistically examine agreement with human ratings and thus did not directly address the substitutability of AI as a rater. Its context was limited to urologic education, leaving generalizability to settings such as prehospital rescue, where dynamics are high and multidisciplinary collaboration is a prerequisite, unclear.

The authors of a previous study targeting junior residents proposed a framework that quantitatively analyzes and assesses NTSs by applying clustering methods to in situ movement paths and conversational data. While the results indicated the possibility of an objective alternative to subjective evaluation and pointed to a new direction for NTS assessment [13], the study authors did not sufficiently evaluate nonverbal and attitudinal aspects within NTSs—such as interpersonal manner—and its targets were confined to in-hospital settings; therefore, generalizability and practicality for prehospital rescue, which requires rapid response under highly variable conditions and limited resources, were insufficiently examined. In addition, the authors of such studies have mainly targeted individual, static outcomes. In unpredictable and dynamic team-based rescue activities, the mechanism by which AI evaluates NTSs remains unknown. Thus, there is a specific gap regarding whether LLMs can validly assess team-level NTSs in prehospital or out-of-hospital emergency simulations using conversation transcripts.

The central question of this study is not only whether AI and human ratings align but also how the quality of their evaluations differs. Our primary hypothesis was that AI-based evaluations would provide ratings of team NTSs that are comparable to those of faculty (paramedic instructors). Our secondary hypothesis was that we would be able to describe the direction and magnitude of score differences between AI and faculty across the five NTS domains. The objective of this study was to evaluate the practical validity of AI-based assessment of NTSs in prehospital rescue simulations by comparing it with professional ratings provided by paramedic instructors using conversation transcripts.

Beyond simply providing another AI-versus-human comparison, in this study, we extend LLM-based NTS assessment into prehospital team simulations of dynamic, time-constrained, and resource-limited out-of-hospital emergency care. In this context, we treat the LLM as a standardized “linguistic lens” applied to conversation transcripts and faculty raters as a “contextual lens” that integrates scene dynamics and professional judgment. By examining where these lenses converge and diverge in prehospital scenarios, we aim to inform a hybrid framework in which AI augments—but does not replace—human-centered NTS assessment.

## 2. Materials and Methods

### 2.1. Study Design and Setting

This study was conducted at Nippon Sport Science University (NSSU, Tokyo, Japan), Yokohama Kenshidai Campus, Clinical Training Room (Yokohama, Kanagawa, Japan), during the scheduled prehospital simulation course in January 2025. Simulations were delivered in small-group sessions using standardized scenarios and a common assessment rubric.

This study was a comparative validation study conducted to examine criterion-related validity by comparing AI-based ratings of NTSs with human raters’ scores. We evaluated five NTS domains that were adapted from an existing faculty rubric, which itself had been developed by drawing on established NTS frameworks such as the Anaesthetists’ Non-Technical Skills (ANTS) system and the TeamSTEPPS team-training model [14,15]. The five domains were as follows: (1) communication and interpersonal manner (tone of voice, eye contact, facial expressions, and appropriate demeanor); (2) order and completeness of information gathering (checking for allergies, current medications, past medical history, and time of last oral intake); (3) level of detail in follow-up questioning (clarifying symptom location, severity, and course over time); (4) context-appropriate actions (scene safety checks, performing necessary assessments, requesting additional support, and making transport decisions); and (5) time management (overall pace, avoidance of unnecessary delay, timing of key interventions). These domains were chosen because they reflect core skills required in prehospital care, namely, communication, information gathering and situation awareness, decision-making, task management, and team coordination under time pressure. The existing NTS ratings provided by faculty raters (paramedic instructors) were treated as the reference standard for validating the AI scores. Simulations were performed in a partitioned activity space within the university’s clinical training laboratory. Audio for evaluation was collected by video recording the simulations with an iPad and extracting the audio track from the video file. We used the STROBE reporting guidelines when drafting this manuscript.

### 2.2. Participants

Eligibility criteria included all first-year paramedic students enrolled in the course during the study period. Exclusion criteria were absence on the simulation day, refusal to participate, and incomplete outcome data. In total, 88 first-year students from the Department of Emergency Medical Science at Nippon Sport Science University, who had received prior lectures on NTSs, were eligible. Of these, 73 ultimately provided written informed consent to participate. For the AI–faculty comparison analyses, we used data from the 64 consenting students who completed the simulation in fixed pairs, yielding 32 two-person rescuer pairs (hereafter, “pairs”) as the primary unit of analysis. Study participation was voluntary; the purpose, procedures, personal data protection, and the right to withdraw at any time were explained in writing and verbally. Although researchers and collaborators were faculty and students, individuals were not personally identifiable; cooperation was voluntary, refusal carried no academic penalty, and all data were de-identified and irreversibly anonymized by assigning new numbers.

### 2.3. Data Collection and Preprocessing

Simulations comprised spectator first aid at a soccer stadium. Each team comprised four persons: two student rescuers, one patient, and one family member or friend. Within each group, the two rescuers worked together as a single clinical pair, and both faculty and AI assigned one set of NTS scores per pair and scenario. Each scenario lasted 5 min; the three chief-complaint scenarios used were chest pain, dyspnea, and dizziness. Scenario type was assigned using a computer-generated simple randomization sequence. Allocation was performed by a study coordinator who was not involved in outcome assessment. Three paramedic raters independently scored the five NTS items using a 3-level rubric (5/3/1 points). Refer to Appendix A for the detailed rubric. Faculty raters were blinded to AI-generated scores and to other raters’ scores. Both AI and faculty applied the same evaluation rubric with identical domain definitions and a three-level ordinal scale. Transcripts were de-identified and processed under a standardized pipeline to reduce information bias. Conversation during the simulation was audio recorded and transcribed verbatim and then organized into a comma-separated values (CSV) file with the following fields: utterance ID, start/end timestamps, speaker (Rescuer A, Rescuer B, Patient, or Associate), gender, and utterance content. Every utterance was categorized into one of four types: instruction (concrete directives to a teammate), explanation (informational statements to the patient), question (history-taking), or emotional expression (reassuring/empathetic phrases). Based on the conversation data, we evaluated NTSs with OpenAI’s ChatGPT-4o (May 2025 release; OpenAI, L.L.C., San Francisco, CA, USA) using the same rubric to derive the AI scores.

### 2.4. AI-Based NTS Evaluation

To ensure reproducibility and standardization, we submitted a structured prompt to ChatGPT-4o for each conversation dataset. We selected ChatGPT-4o for pragmatic reasons. At the time of data collection, it was one of the widely available large language models with reliable support for Japanese and stable access via the web interface, which allowed us to run all evaluations without custom infrastructure. ChatGPT-4o has strong general instruction-following capabilities, and we therefore judged it suitable for generating rubric-based, structured outputs (5/3/1-point scores for each domain plus brief textual justifications) from a single prompt. In addition, ChatGPT-based systems have been the focus of prior medical and medical-education research; thus, using GPT-4o situated this study within the emerging literature on LLM-supported assessment. To minimize nondeterminism, each analysis involved the use of the temporary chat (no session history) mode so that past interactions could not bias scoring and each case would be evaluated independently from a clean state. We then created paired datasets aligning faculty and AI scores and compared them. AI analyses were performed in isolated sessions without access to faculty ratings or any identifying information to minimize information leakage.

LLM configuration (original runs): We used ChatGPT (web UI) to obtain AI scores. In the web interface, sampling parameters such as temperature, top_p, and output-length limits are not user-configurable or documented; thus, these parameters are unspecified for the present analyses. All conversation transcripts and AI outputs were in Japanese, and we accessed ChatGPT-4o via the Japanese-language interface. The model/version was GPT-4o (May 2025 release), and the user prompts (with an English translation for reference) are provided in Appendix A.

Replication protocol (not used in the present analyses): For future replication via the OpenAI API, a deterministic configuration can be used: temperature = 0.0, top_p = 1.0, and max_output_tokens = 256, together with the fixed prompt in Appendix A. This protocol is offered to facilitate independent reproduction without modifying the results reported herein.

### 2.5. Outcomes

The primary outcome, corresponding to our primary hypothesis that AI-based ratings would be comparable to faculty ratings, was the agreement between AI and faculty scores in each NTS domain. Agreement was quantified using weighted Gwet’s AC2 (quadratic weights) with 95% bootstrap confidence intervals, and we pre-specified AC2 ≥ 0.60 as the adequacy criterion for substantial agreement. Secondary outcomes, corresponding to our secondary hypothesis regarding score differences, were domain-wise paired differences between AI and faculty scores, analyzed with Wilcoxon signed-rank tests (five tests, Holm-adjusted *p*-values) and Bayes factors quantifying evidence supporting or rejecting the null hypothesis of no difference.

### 2.6. Statistical Analysis

Descriptive statistics for ordinal NTS scores and other non-normally distributed variables are reported as median and interquartile range (median [IQR]). Approximately normally distributed continuous variables (e.g., age) are summarized as mean and standard deviation (mean ± SD). Because the study was embedded in a required course, the maximum feasible sample size was the full class cohort (*n* = 88), of whom 64 students (32 fixed pairs) contributed complete simulation data for the AI–faculty comparison analyses. We did not perform a priori power calculation; instead, we report 95% bootstrap confidence intervals for agreement coefficients and Bayes factors to indicate the precision achievable with this sample. All comparisons of AI and faculty scores and all agreement statistics (Gwet’s AC2 and weighted κ) were conducted at the pair level (*n* = 32), with one AI–faculty score pair per domain and rescuer pair; individual student characteristics (*n* = 64) are reported descriptively in Table 1 only. As a simple sensitivity analysis with respect to sample size, we interpreted the widths of the 95% bootstrap confidence intervals for AC2 (Table 2) and the magnitudes of BF10 as indicators of how precisely agreement and score differences could be estimated with the available 32 teams. We first assessed the normality of the score data using the Shapiro–Wilk test and then compared AI and faculty scores with Wilcoxon signed-rank tests (paired, two-sided).

The *p*-values for the five domain-wise tests were adjusted for multiple comparisons using Holm’s sequentially rejective procedure [16]; two-sided α = 0.05 after Holm correction defined statistical significance. To address limited statistical power from a relatively small sample, we complemented frequentist tests with Bayesian analysis: using a method equivalent to a paired *t*-test, we computed Bayes factors (BF10), evaluating evidence as strong for a difference if BF10 > 3, inconclusive if 1/3 ≤ BF10 ≤ 3, and strong for no difference if BF10 < 1/3. We also performed Bayesian estimation of the mean AI–faculty score difference and its 95% credible interval (CrI); CrIs excluding 0 were interpreted as indicating a substantive difference. We treated Holm-adjusted *p*-values and Bayes factors as complementary indices rather than two independent tests, basing our main inferences on cases where they converged in the same direction and regarding discordant results as inconclusive. Analyses were conducted in Python 3.12.12 (Python Software Foundation, Wilmington, DE, USA) using the following open-source libraries: PyMC 5.26.1, ArviZ 0.22.0, pandas 2.2.2, and NumPy 2.0.2. Bayesian estimation and Bayes factors were computed with PyMC and ArviZ, whereas frequentist tests were performed in IBM SPSS Statistics 29 (IBM Corp., Armonk, NY, USA).

For agreement, to mitigate the “kappa paradox [17]” due to marginal distributions and prevalence effects, we used weighted Gwet’s AC2 as the primary index for the three-level ordinal scale, adopting quadratic weights. Of note, 95% confidence intervals (CIs) for AC2 were obtained by means of bootstrap resampling (1000 replicates). AC2 values can theoretically range from –1 (complete disagreement), through 0 (chance-level agreement), to 1 (perfect agreement); values below 0 indicate agreement below chance, meaning that the two sets of ratings tend to diverge systematically rather than merely by random fluctuation. Values below roughly 0.40 are generally interpreted as indicating at most fair agreement, values around 0.41–0.60 are generally interpreted as moderate, and values above 0.60 are generally interpreted as substantial agreement, following commonly cited guidelines for κ-like coefficients [18]. In this study, we therefore treated AC2 ≥ 0.60 as a threshold for substantial agreement between AI and faculty ratings. Weighted Cohen’s κ (range −1 to 1) was also calculated as a secondary index and interpreted using the same qualitative categories, with κ ≥ 0.60 likewise regarded as indicating substantial inter-rater agreement. Data handling additionally used pandas and NumPy. There were no missing outcome data; all enrolled participants with complete AI and faculty ratings were included in the analyses without imputation.

We used ChatGPT and GPT-5 (accessed October 2025) in a limited way to support code drafting and language editing (first-pass translation from Japanese and refinement of non-technical text). All statistical analyses were implemented, verified, and interpreted by the authors; AI tools were not used to execute analyses, generate figures, or create references, and the authors take full responsibility for the accuracy and integrity of the manuscript.

## 3. Results

Of the 88 eligible first-year paramedic students, 73 provided consent (83%); 6 were absent on the simulation day; and 9 declined to participate. The analysis set comprised 64 students (32 pairs) who completed the simulation. Participant characteristics are summarized in Table 1. The mean (SD) age was 18.8 (0.4) years; 17 participants (26.6%) were female and 47 (73.4%) were male. There were no missing values for age or sex.

In two-sided Wilcoxon tests with Holm correction, AI scores significantly exceeded faculty scores in three NTS domains—communication and interpersonal manner (*p* < 0.001), context-appropriate actions (*p* < 0.001), and time management (*p* < 0.001); the corresponding median differences were +1.4, +1.6, and +1.3 points, respectively. In contrast, the domains of order and completeness of information gathering and detail of follow-up questioning showed no significant differences between AI and faculty ratings. Comparisons between AI and faculty ratings across domains are summarized in Table 2.

Bayes factors (BF10) likewise provided strong evidence for differences in the same three domains (22.4 for communication and interpersonal manner; 37.1 for context-appropriate actions; 23.4 for time management; Table 2). Conversely, order and completeness of information gathering (BF10 = 1.7) and detail of follow-up questioning (BF10 = 0.3) yielded insufficient evidence; mean differences (95% credible intervals) were −0.495 (−0.981 to 0.095) and +0.059 (−0.526 to 0.769), respectively, both spanning zero.

Agreement, quantified using weighted Gwet’s AC2 (quadratic weights), did not reach 0.60 in any domain. AC2 values (95% CIs) were as follows: communication and interpersonal manner, −0.152 (−0.340 to 0.079); order and completeness of information gathering, 0.194 (−0.040 to 0.476); detail of follow-up questioning, 0.297 (0.017 to 0.553); context-appropriate actions, −0.153 (−0.328 to 0.062); and time management, 0.091 (−0.177 to 0.326). In the domains of communication and interpersonal manner and context-appropriate actions, we observed small negative AC2 values, indicating agreement below chance. Although only the lower bound for follow-up detail exceeded 0, none approached the operational threshold of 0.60; weighted Cohen’s κ likewise remained < 0.60 across all domains.

Accordingly, the primary hypothesis—that (i) there would be no score differences across all five domains and (ii) each domain would achieve AC2 ≥ 0.60—was not supported. Although two domains showed no significant difference, both AC2 and κ remained < 0.60 throughout.

## 4. Discussion

In this study, we examined the practical validity of AI-based evaluation of non-technical skills (NTSs) by comparing assessments derived from conversation data in prehospital rescue activities with professional ratings provided by paramedic evaluators.

Our primary hypothesis that AI-based ratings would be comparable to faculty ratings was not supported. Although AI and faculty reached similar median scores in two domains (order and completeness of information gathering and detail of follow-up questioning), AI showed a statistically strong tendency to assign higher median scores than human instructors in three domains: communication and interpersonal manner, context-appropriate actions, and time management. We posited that there was a fundamental difference in evaluative frameworks: whereas AI is adept at capturing the formal and structural aspects of language, human instructors make integrated judgments of contextual and practical aspects—including on-scene conditions and nonverbal cues—such that a “qualitative divergence in evaluation” exists.

The weighted Gwet’s AC2 indicating agreement between AI and human raters remained < 0.60 for all items, despite these higher AI scores in three domains.

These results are thought to stem from AI’s tendency to readily detect elements amenable to normative and quantitative evaluation in conversation content. For example, clarity of utterances, completeness of information provision, and appropriately timed directives can be analyzed using natural language processing methods [19].

AI readily recognizes grammatical correctness and formal linguistic features—such as word order and syntactic patterns—and classifies and evaluates utterances based on these elements [20,21]. With respect to “communication and interpersonal manner,” it is plausible that AI focused on objective elements such as the use of honorifics, affirmative phrasing in students’ utterances, and the number of turns taken. While such formal linguistic features are important as a form of consideration toward patients and their associates, human raters, for their part, make more comprehensive judgments that include nonverbal aspects such as facial expression, gaze, and prosody; their vantage point thus likely differed from that of AI [22,23,24].

In this study, to account for the “kappa paradox,” we adopted Gwet’s AC2 as the principal agreement index. AC2 failed to reach 0.60 for all five items, and the auxiliary index of weighted κ likewise remained below 0.60. The low agreement observed in this case is thought to represent a structural divergence arising from systematic differences in criteria between AI and human raters—for example, AI relatively emphasizes linguistic completeness and information quantity, whereas human raters emphasize clinical appropriateness and triage-driven prioritization.

Beyond differences in how linguistic and contextual information are weighted, at least two additional factors may have contributed to the observed divergences. First, faculty raters may exhibit halo effects. For example, when a scenario includes incomplete assessment or clinically questionable decisions, faculty may deliberately avoid assigning high NTS scores in other domains as well, resulting in overall stricter ratings. In contrast, the LLM scores each domain independently, based solely on the transcript content. Second, the three-level rating scale used in this study has limited resolution. Consequently, even small differences in judgment between AI and faculty can more easily cross category boundaries, which may magnify apparent discrepancies in scores and lead to lower agreement coefficients.

At the root of these “different evaluative criteria” lies the symbol grounding problem [25,26]. Large language models (LLMs) learn formal and structural patterns of language from massive text corpora. However, the symbol (word) “thank you” handled by an LLM is not tied to “grounded meaning” in the real world—such as heartfelt facial expressions of gratitude, tone of voice, and situational context. As proposed by Harnad (1990) in cognitive science, the “symbol grounding problem” suggests that AI evaluation may remain at the level of surface linguistic expression and fail to capture deeper nuances, such as the true intentions and emotions behind it [25]. In this study, the fact that AI rated “communication and interpersonal manner” more highly is thought to be because it detected formal and structural features such as the frequency of honorifics and the completeness of utterances. Human raters, however, also include in their evaluations “appropriateness under pressure” in the situations where the utterances are made and “genuine empathy” conveyed by nonverbal information such as tone of voice and gaze. It is precisely this evaluation of “grounded meaning,” which AI cannot capture, that constitutes the essential factor generating the divergence.

Operationally, this distinction maps onto our five NTS domains. Constructs such as “communication and interpersonal manner” and “context-appropriate actions” depend strongly on grounded meaning—nonverbal affect, attunement to scene dynamics, and value-laden judgments—whereas “order and completeness of information gathering” and “detail of follow-up questioning” can be evaluated largely from the linguistic record. The symbol grounding gap therefore offers a concrete explanatory mechanism for the pattern we observed, in which AI tended to assign higher scores yet AC2/κ remained below 0.60, particularly in socio-emotional domains; an empirical hypothesis for future studies is that AI–human discrepancies and low agreement coefficients should be most pronounced in domains that rely heavily on grounded meaning and should decrease if multimodal (audio/video) data are incorporated.

Evaluation by human raters does not stop at judging the content of individual utterances. Raters integrate whether team members share situation awareness smoothly and coordinate effectively—that is, shared mental models (SMMs)—in their judgments [27]. Their evaluations do not remain at the analysis of individual utterances but assess higher-order, team-level states such as the degree to which situation awareness and action plans are shared among team members [27,28]. This is a context-dependent, holistic judgment that exceeds the capacity to process individual linguistic symbols and clearly indicates present limits of AI-based evaluation. In short, the fundamental difference in cognitive frameworks—“AI, which remains at the processing of individual symbols,” versus “humans, who evaluate grounded meanings and shared context”—is considered the essential mechanism producing the evaluative divergence observed in this study.

From an operational standpoint in clinical education, this symbol grounding gap constrains how AI-generated NTS scores should be used. At present, scores from a transcript-only model should not serve as stand-alone criteria for high-stakes decisions such as pass/fail or promotion, because constructs such as empathy, attitude, and trust-building depend on nonverbal and contextual cues outside the model’s view. There is a risk that superficially polite but clinically weak interactions are overrated, whereas concise yet context-appropriate communication under pressure is undervalued. We therefore envisage near-term use mainly as low-stakes, formative support—for example, screening large numbers of transcripts and flagging segments for human review—within quality assurance processes where final judgments remain with trained educators.

In a double-blind study by Pears et al., experts evaluated annotated transcribed text and compared AI and human feedback styles, suggesting the effectiveness of role sharing between AI and humans. Our study complements those results by presenting a measurement constraint: although AI consistently assigns higher scores, it lacks sufficient agreement to serve as a substitute for human evaluation.

Nevertheless, even in the three items in which BF10 far exceeded 10—22.4 (communication and interpersonal manner), 37.1 (context-appropriate actions), and 23.4 (time management)—and strong evidence for the superiority of AI evaluation was obtained, caution is warranted: this apparent advantage is highly likely to rely primarily on linguistic/formal features, and may fail to capture nonverbal cues and context-dependent judgments adequately. For example, nonverbal/attitudinal elements such as “interpersonal manner” and “empathetic engagement” cannot be accurately grasped by current AI systems. Human raters infer attitude and emotional consideration from elements such as students’ facial expressions, tone of voice, and gaze; AI evaluation based solely on conversation data may fail to capture nonverbal aspects, leaving important decision-making materials insufficient. AI excels at evaluations based on linguistic patterns and temporal indices, whereas it has limits with respect to uniquely human social cognition and affective judgment.

This study is meaningful in that the results suggest the possibility that AI-based NTS evaluation can capture perspectives different from those of human evaluation; concurrently, because agreement (AC2/κ) remained below 0.60 across all domains, it is not possible to conclude that AI can be introduced immediately as an “objective and consistent assessment method.” Operational benefits such as personalized education, reduced faculty burden, and instant feedback remain theoretical possibilities and were not directly evaluated in this study; therefore, future prospective research is necessary to verify their effects.

Conversely, AI has limitations in its ability to evaluate nonverbal and socio-emotional aspects, and it has not yet reached the point of accurately capturing essential NTS components such as “empathy,” “attitude,” and “trust-building.” Our results also suggest that AI tends to emphasize formal correctness, whereas humans may incorporate contextual elements such as on-scene adaptability and interpersonal subtleties into their evaluations. Accordingly, rather than using AI-based evaluation alone, the design and validation of hybrid evaluation models for use in combination with human evaluation represent a reasonable direction going forward.

Moreover, future systems will likely need to move beyond text-only inputs. In prehospital simulations, multimodal incorporation of audio and video data—such as facial expression, gaze, prosody, and body movement—could enable AI models to approximate socio-emotional and interpersonal skills (e.g., empathy, attitude, trust-building) more directly, rather than inferring them from transcripts alone. Such multimodal models would need to be developed and validated under strict privacy and governance frameworks, and under such circumstances, we anticipate that AI-based NTS evaluation will function best not as a replacement for human raters, but as a partner supporting them.

Considering our results, there is an implied need for the authors of future studies to examine an AI-assisted debriefing framework in which time-series-based probabilistic state estimation (e.g., dynamic Bayesian networks) and explainable AI methods are used to generate interpretable evidence displays that support human-led debriefing [29,30]. For example, an AI system could highlight specific time points at which shared situational awareness deteriorated (e.g., when vital-sign sharing lapsed) together with textual explanations that instructors and learners can use as a “cognitive mirror” during reflection. Although reinforcement-learning-based personalization is also conceivable as a way to adapt feedback to individual learners, confirmation of its effectiveness and practical feasibility constitutes a separate research agenda [31]. For the effective practice of future NTS education, emphasis is required not only on the development of evaluation methods but also on nonverbal aspects within the educational content itself. Evaluators should take the standpoint of patients and their associates, demonstrate an attitude of empathetic understanding, and explicitly teach nonverbal communication—such as facial expressions, gaze, and prosody—necessary for building trust. These elements are not matters that can be taught merely as “skills,” but attitudes and values that should be cultivated through experiential and iterative learning. In parallel with the refinement of evaluation models enabling collaboration between AI and humans, more systematic examination is required of approaches to educational practice that focus on nonverbal and socio-emotional aspects.

This study has several limitations. First, both the study participants and the raters were Japanese. The results of previous studies have shown that respondents in East Asia show a relatively strong tendency toward a “moderacy response style”—preferring midpoints and avoiding extreme values on Likert scales [32]—which may have affected the rating distributions and agreement indices. We evaluated only a single LLM (ChatGPT-4o); although it was chosen as a representative high-performing model for the aforementioned reasons, the extent to which our findings generalize to other architectures and vendors remains uncertain. Furthermore, all prompts and conversation transcripts in this study were written in Japanese, and GPT-4o was accessed via the Japanese-language interface. Because Japanese does not explicitly mark word boundaries and expresses politeness and empathy through honorific forms and formulaic phrases, the model’s internal tokenization and Japanese-specific cultural–linguistic priors may have led it to overweight surface politeness cues in the “communication and interpersonal manner” domain. Additionally, all simulations were conducted within a single Japanese paramedic training program with first-year students, where educational and organizational norms—such as strong emphasis on formal politeness, deference to senior staff, and adherence to protocols—are likely to shape both student behaviors and faculty scoring practices. These cultural and training-system factors may influence which aspects of communication, teamwork, and risk management are emphasized; therefore, the magnitude and direction of AI–human score discrepancies observed in this study may differ in other EMS systems, professions, training stages, or countries. The generalizability of the present findings to other cultures and ethnic groups should therefore be judged with caution. Second, the analytic sample was limited to 32 two-person teams (64 students) from a single cohort, which constrains the precision and generalizability of the agreement and Bayes factor estimates; the findings should therefore be interpreted with appropriate caution. Third, the AI evaluations were based solely on transcribed conversation data and did not capture important nonverbal information—such as tone of voice, facial expressions, and gestures—that instructors would likely have included in their ratings. This is a fundamental limitation arising from the “symbol grounding problem.” Fourth, the AI evaluations used in this study depended on a specific prompt, and the prompt design may have influenced the results. Fifth, although we used a temporary chat function to suppress AI nondeterminism in order to secure reproducibility, further refinement of the evaluation algorithm is required for stable operation in real educational settings. In addition, AI output contains black-box elements that pose challenges to transparency and explainability in evaluation; future implementation will require an understanding of—and countermeasures for—these characteristics. Finally, the evaluation scenarios were limited to three scenario types, which is also a limitation when examining the generalizability of AI evaluation to diverse rescue situations.

## 5. Conclusions

In this study, our results demonstrated that, in prehospital rescue simulations characterized by high variability and the need for rapid responses, it is not yet appropriate to regard AI as a stand-alone substitute for NTS assessment. Although AI exhibited certain detection capabilities based on the formal and structural features of conversation, its lack of integration of nonverbal information and team context meant that substantial agreement with human evaluations could not be confirmed; thus, it does not serve as a replacement for evaluators’ holistic judgments. Such divergence may reflect not only the absence of nonverbal integration and insufficient calibration of evaluation criteria but also differences in symbol grounding and shared mental models. Looking ahead, it will be necessary to evaluate a hybrid operation that combines AI’s data-processing capacity with humans’ contextual understanding and educational expertise, positioning AI as a support tool to strengthen human evaluation and debriefing. The present findings provide an important theoretical and empirical basis for determining the appropriate role of AI in next-generation simulation-based education.

## Figures and Tables

**Table 1:** Baseline characteristics of participants (*n* = 64; 32 pairs).

Variable	Summary		Missing
Age (years), mean (SD)	18.8 (0.4)		0
Sex, *n* (%)	Female	17 (26.6)	0
	Male	47 (73.4)	0

Values are summarized at the individual student level. Values are *n* (%) unless otherwise indicated. Continuous variables are summarized as the mean (SD). Abbreviations: SD, standard deviation.

**Table 2:** Comparison between AI and paramedic instructors across five non-technical skill domains (*n* = 32 pairs).

Domain	*n* (Pairs)	AI, Median (IQR), Point	Faculty, Median (IQR), Point	Z	P (Holm)	Median Difference (AI-Faculty), Point	BF10	Bayesian Mean Difference (AI-Faculty), Point (95% CrI)	AC2 (95% CI)	κ (95% CI)
Communication and interpersonal manner	32	5.0 (5.05.0)	3.0 (3.0–3.0)	3.572	<0.001	1.4	22.4	1.429 (0.775–2.049)	−0.152 (−0.340–0.079)	−0.038 (−0.187–0.112)
Order and completeness of information gathering	32	3.0 (1.5–3.0)	3.0 (1.0–5.0)	1.886	0.120	−0.5	1.7	−0.495 (−0.981–0.095)	0.194 (−0.040–0.476)	0.409 (0.211–0.607)
Detail of follow-up questioning	32	3.0 (3.0–3.0)	3.0 (1.5–3.0)	0.206	0.837	0.1	0.3	0.059 (−0.526––0.769)	0.297 (0.017–0.553)	−0.136 (−0.441–0.170)
Context-appropriate actions	32	5.0 (3.5–5.0)	3.0 (3.0–3.0)	4.185	<0.001	1.6	37.1	1.625 (1.075–2.114)	−0.153 (−0.328–0.062)	0.008 (−0.135–0.152)
Time management	32	5.0 (3.0–5.0)	3.0 (3.0–3.0)	3.632	<0.001	1.3	23.4	1.254 (0.691–1.797)	0.091 (−0.177–0.326)	−0.011 (−0.202–0.181)

Analyses were conducted at the pair level (*n* = 32), with one AI–faculty score pair per domain and rescuer pair. Z, *p*: Two-sided Wilcoxon signed-rank tests (AI vs. Faculty); *p*-values (five tests) were Holm-adjusted. BF10: JZS prior (r = √2/2). Values > 10 indicate “strong evidence.” Scores and score differences (median and Bayesian mean) are measured in points on the 3-level rubric (5/3/1). Gwet’s AC2 (quadratic weights) 95% CIs were estimated via bootstrap with 1000 resamples; weighted κ CIs via the asymptotic method. Agreement coefficients (AC2 and κ) range from −1 (perfect disagreement) to 1 (perfect agreement); negative values indicate agreement below chance (i.e., systematic disagreement).

## Data Availability

The data supporting the findings of this study contain personally identifiable or sensitive information from student participants and cannot be shared publicly. De-identified data may be available from the corresponding author upon reasonable request and subject to institutional and ethical approvals.

## Abbreviations

The following abbreviations are used in this manuscript:

AI	artificial intelligence
NTS	non-technical skills
AC2	agreement coefficient 2 (Gwet’s AC2)
BF10	Bayes factor in favor of the alternative hypothesis
IQR	interquartile range
SD	standard deviation
LLM	large language model
CSV	comma-separated values

## Appendix A. AI Evaluation Prompt

You are an objective and consistent evaluator of conversation data from prehospital care scenarios.

Please carry out the following Tasks 1–4 as a fresh evaluation, without relying on any previous judgments.

The target data are conversation transcripts from prehospital care, provided in CSV format.

Task 1: Load and review the conversation data

Load the CSV file with the following column structure:

- Column A: Utterance ID
- Column B: Start time of the utterance
- Column C: End time of the utterance
- Column D: Speaker
  ○ a: Rescuer A
  ○ b: Rescuer B
  ○ c: Patient
  ○ d: Bystander/related person

- Column E: Gender
- Column F: Utterance content

After loading the data, check that the structure is correct and report the following information in bullet points:

1. Total number of utterances.
2. Types of speakers who appear and how many speakers of each type.
3. A few sample utterances (2–3 examples, with utterance ID and utterance content).

Task 2: Classify each utterance into one of four categories

Classify every utterance into one of the following four categories:

1. Instruction: Concrete directions given to another team member (e.g., “Check the blood pressure,” “Prepare the stretcher”).
2. Explanation: Explanations or information provided to the patient or bystanders about the situation, condition, or procedures.
3. Question: Questions addressed to the patient or bystanders (including SAMPLE questions and other history-taking).
4. Emotional support/empathy: Utterances that provide reassurance, empathy, or emotional support (e.g., building trust, reducing anxiety).

Output format: Provide the results as a Markdown table with the following three columns:

Utterance ID|Utterance content|Category

- The utterance content must be reproduced verbatim, exactly as in the transcript.
  ○ Do not summarize, shorten, or paraphrase.

Task 3: Rate team performance on five items using a 3-level (5/3/1) scale

Evaluate the team’s performance on the following five non-technical skill (NTS) domains using a 3-level rubric (5, 3, or 1 point).

For each item, you must provide:

- A score (5, 3, or 1).
- The basis for the evaluation, including specific quoted utterances as evidence.
- Points for improvement, in the form of concrete advice that can lead to better behavior.

Five evaluation items and criteria:

1. Communication and interpersonal manner

○ 5 points: Tone of voice, eye contact, expressions, and overall demeanor are appropriate and natural, and they provide reassurance and a sense of safety to the patient and bystanders.
○ 3 points: Some instability or minor issues are present, but overall, the interaction maintains trust and is acceptable.
○ 1 point: There are noticeable behaviors that could create anxiety, distrust, or discomfort for the patient or bystanders.

2. Order and completeness of information gathering

○ 5 points: The rescuer systematically asks about key elements such as symptoms, allergies, medications, past medical history, last oral intake, and events leading to the incident in a logical order and covers all necessary items.
○ 3 points: The order of questions is somewhat disorganized, but the rescuer mostly covers the necessary items.
○ 1 point: There are major omissions in important items, or the questioning is highly disorganized.

3. Level of detail in follow-up questioning

○ 5 points: As needed, the rescuer asks follow-up questions in a focused and concrete way about aspects such as location, severity, and time course of symptoms.
○ 3 points: Follow-up questions are asked, but they are relatively superficial or lack depth, leaving some important details unexplored.
○ 1 point: Follow-up questioning is minimal or absent, or remains at a very superficial level.

4. Context-appropriate actions (choice of actions suited to the situation)

○ 5 points: Based on the patient’s condition and scene context, the rescuer selects high-priority observations, interventions, and communications at appropriate times (e.g., safety checks, necessary assessments, calls for backup or transport decisions).
○ 3 points: There are no major problems, but there are some opportunities to improve prioritization or timing of actions.
○ 1 point: Important assessments or interventions are omitted, delayed, or replaced by less appropriate actions.

5. Time management

○ 5 points: Within a limited time, the rescuer efficiently completes necessary questioning, observation, and interventions, maintaining an appropriate pace.
○ 3 points: Overall tasks are completed, but there are some inefficient parts (e.g., spending too long on certain questions or explanations).
○ 1 point: Time allocation is inappropriate, and the rescuer fails to devote sufficient time to important tasks.

Output format: Provide the results as a Markdown table with the following four columns:

Evaluation item|Score (5/3/1)|Basis for the evaluation (including quoted utterances)|Points for improvement

- In the “Basis for the evaluation” column, include utterance IDs and verbatim quotes as evidence.

Task 4: Write a feedback comment addressed to the rescue team

Based on the results of Task 3, write a feedback comment for the rescuers.

Follow the conditions and structure below, and use polite, constructive, and encouraging language.

Conditions:

- For each evaluation item, include both “strengths” (what went well) and “areas for improvement.”
- Briefly quote relevant utterances where appropriate and provide actionable advice that can help improve future behavior.
- The total length should be approximately 300–500 Japanese characters, concise but covering all key points.

Suggested structure:

- Introduction: Overall impression and general summary of performance.
- Body: Specific feedback for each evaluation item (strengths and areas for improvement).
- Conclusion: Positive, forward-looking message expressing expectations for future improvement.

Global reproducibility constraints (apply to all tasks)

- Apply the above rating criteria and rules exactly as written.

○ Do not introduce your own criteria or modify the rubric.

- Base all judgments solely on the content of the conversation data provided.

○ Do not rely on outside knowledge or imagination beyond what is explicitly stated.

- When quoting utterances, use the original text verbatim from the transcript.

○ Do not summarize or paraphrase quoted utterances.

- All outputs (tables and comments) must be written in Japanese.
